# Cooking and Digestion

**Published:** 1892-08

**Authors:** 


### COOKING AND DIGESTION.

Experiments as to the comparative digestibility of various foods, cooked and uncooked, tend to show that boiled fish is less digestible than boiled beef, but many kinds of fish are as digestible as veal, mutton or fowls. Dr. M. Popoff comes to the following conclusions :—
1. Beef as well as fish is better digested raw than cooked. Cooking diminishes digestibility ; its influence is more marked on beef than on fish. 2. The longer the cooking the worse the digestion. 3. Beef (except smoked) is generally better digested than fish, when cooked in the same manner. 4. The smoking of fish facilitates peptonization. Smoked fish is more digestible than raw or cooked. On the other hand, beef smoked is more difficult to peptonize than in any other condition. 5. It is worthy of mention that the fat of fish does not hinder digestibility ; its disintegrating action seems to cause fatty fish to be digested more easily than lean fish.

